# The Cytogenetic Landscape of Pediatric Chronic Myeloid Leukemia Diagnosed in Chronic Phase

**DOI:** 10.3390/cancers14071712

**Published:** 2022-03-28

**Authors:** Axel Karow, Gudrun Göhring, Stephanie Sembill, Friederike Lutterloh, Fina Neuhaus, Sara Callies, Elke Schirmer, Zofia Wotschofsky, Oisin Roche-Lancaster, Meinolf Suttorp, Manuela Krumbholz, Markus Metzler

**Affiliations:** 1 Department of Pediatrics and Adolescent Medicine, Friedrich-Alexander-Universität Erlangen-Nürnberg (FAU), D-91054 Erlangen, Germany; stephanie.sembill@uk-erlangen.de (S.S.); elke.schirmer@uk-erlangen.de (E.S.); zofia.wotschofsky@uk-erlangen.de (Z.W.); manuela.krumbholz@uk-erlangen.de (M.K.); markus.metzler@uk-erlangen.de (M.M.); 2 Comprehensive Cancer Center Erlangen-EMN (CCC ER-EMN), D-91054 Erlangen, Germany; oisin.roche-lancaster@uk-erlangen.de; 3 Department of Human Genetics, Hannover Medical School, D-30625 Hannover, Germany; goehring.gudrun@mh-hannover.de (G.G.); lutterloh.friederike@mh-hannover.de (F.L.); neuhaus.fina@mh-hannover.de (F.N.); callies.sara@mh-hannover.de (S.C.); 4 Center of Medical Information and Communication Technology, University Hospital Erlangen, D-91054 Erlangen, Germany; 5 Medical Faculty, Pediatric Hematology and Oncology, Technical University, D-01307 Dresden, Germany; meinolf.suttorp@uniklinikum-dresden.de

**Keywords:** chronic myeloid leukemia, pediatric chronic myeloid leukemia, Philadelphia chromosome, variant translocations, additional chromosomal aberrations, complex karyotype, tyrosine kinase inhibitor treatment, cytogenetic response, molecular response

## Abstract

**Simple Summary:**

Philadelphia chromosome-positive chronic myeloid leukemia (CML) is characterized by the translocation of the chromosomes 9 and 22. Additional non-Philadelphia aberrations of chromosomes (nPhAs) and their prognostic relevance for the disease course are comparably well known in adult patients with CML. However, due to the rarity of CML in children and adolescents, nPhAs have hardly been determined systematically in these age groups. Here, we present a large analysis of nPhAs detected in a cohort of 161 patients younger than 18 years who had been diagnosed with CML in chronic phase and enrolled in the German national CML-PAED-II registry. We found a distinct distribution of nPhAs in this pediatric cohort with possible impact on treatment response whereas the survival remained unaffected. Our findings emphasize differences in the disease biology between pediatric and adult patients and prompt further joint international efforts to acquire more data on the disease in this age group.

**Abstract:**

Philadelphia chromosome-positive chronic myeloid leukemia (CML) is cytogenetically characterized by the classic translocation t(9;22)(q34;q11), whereas additional non-Philadelphia aberrations (nPhAs) have been studied extensively in adult patients with CML, knowledge on nPhAs in pediatric patients with CML is still sparse. Here, we have determined nPhAs in a cohort of 161 patients younger than 18 years diagnosed with chronic phase CML and consecutively enrolled in the German national CML-PAED-II registry. In 150 cases (93%), an informative cytogenetic analysis had been performed at diagnosis. In total, 21 individuals (13%) showed nPhAs. Of these, 12 (8%) had a variant translocation, 4 (3%) additional chromosomal aberrations (ACAs) and 5 (3%) harbored a complex karyotype. Chromosome 15 was recurrently involved in variant translocations. No significant impact of the cytogenetic subgroup on the time point of cytogenetic response was observed. Patients with a complex karyotype showed an inferior molecular response compared to patients carrying the classic translocation t(9;22)(q34;q11), variant translocations or ACAs. No significant differences in the probability of progression-free survival and overall survival was found between patients with nPhAs and patients with the classic Philadelphia translocation only. Our results highlight the distinct biology of pediatric CML and underline the need for joint international efforts to acquire more data on the disease pathogenesis in this age group.

## 1. Introduction

The Philadelphia chromosome, characterized by the reciprocal balanced translocation t(9;22)(q34;q11), is the pathognomonic hallmark of chronic myeloid leukemia (CML) [1]. A proportion of individuals with CML show additional non-Philadelphia aberrations (nPhAs) already at diagnosis or as a result of clonal evolution during disease progression. The designation nPhAs will be used hereinafter as an umbrella term encompassing the three categories of variant translocation, additional chromosomal aberration (ACAs) and complex karyotype as defined by ≥3 chromosomal aberrations including at least one structural aberration. Synonyms of nPhAs found in the current literature are additional cytogenetic abnormalities, additional cytogenetic findings or clonal cytogenetic aberrations.

In adult cohorts with CML, the frequency, the distribution patterns, and the prognostic implications of nPhA have been well investigated in a considerable number of studies [2,3,4,5]. These have found that nPhAs occur in 10 to 12% of adult cases with chronic phase (CP) CML at diagnosis and bear differential prognostic value depending on the type of aberration. 

Chronic myeloid leukemia in childhood and adolescence is a rare disease with an annual incidence of 1 and 2.2 per million, respectively [6]. Some recent studies have suggested diverse genetic and phenotypic features in pediatric CML [7,8,9,10,11,12,13,14]. However, there is still limited knowledge on potential differences of the cytogenetic features between pediatric and adult patients with CML. To date, there is only one previous analysis by Millot et al. from the International Registry for CML in Children and Adolescents (I-CML-Ped study) addressing the incidence of nPhAs and their potential role in a large group of pediatric patients with CML [15].

The goal of our study was to assess the population-based incidence of nPhAs and the distribution of cytogenetic subgroups among pediatric patients diagnosed with CP CML and consecutively enrolled in the German national CML-PAED-II study and following registry. In addition, we aimed to describe the landscape of nPhAs at diagnosis and assess their impact on cytogenetic and molecular response characteristics and progression-free and overall survival in this cohort of young patients with CML. 

## 2. Patients and Methods

### 2.1. Study Design

This study was based on the data acquired through the CML-PAED-II study [16] and the subsequent registry. The CML-PAED-II study was a prospective, investigator-initiated, academic, multicenter, open-label, single-arm, phase-III clinical trial and assessed the effects of up-front imatinib-treatment in pediatric patients with chronic phase CML. The protocol was conducted in accordance with the Declaration of Helsinki and approved by the institutional ethics boards (EK282 122 006 and EK 236_18 B), registered at EUDRACT (2007-001339-69) and Clinical-Trials.gov (NCT00445822). Informed consent was obtained from the patients’ legal guardians and, if applicable, the patients, after they were provided age-appropriate oral and written information. Data were collected from participating centers using standardized forms at diagnosis and every 3 months thereafter.

### 2.2. Patients

Children and adolescents aged 0–17 years diagnosed with chronic phase CML between 1 January 2006 and 20 November 2020 were eligible. Diagnoses were confirmed by central reference review as per the current guidelines of the World Health Organization [17] and the criteria of the European LeukemiaNet (ELN) [18].

One patient with ACA (UPN 14) and one patient with complex karyotype (UPN 17) had been included in the previous analysis from the I-CML-Ped study [15]. Patients presenting with de novo blast phase (BP) CML were excluded from the study based on the differences concerning the disease biology and the initial treatment regimen in this group of patients described earlier [19].

### 2.3. Laboratory Methods

Laboratory analyses were conducted exclusively in certified laboratories. 

Chromosome G or fluorescence R-banding analysis was performed on short-term cultures (24–48 h) of bone marrow (BM) aspirates. Cell cultivation, chromosome preparation and fluorescence-R banding were performed as previously described [20,21]. A total of 20 metaphases were analyzed when possible and the karyotypes were described according to the International System for Chromosome Nomenclature (ISCN, 2016).

*BCR::ABL1* transcript types and levels in the peripheral blood (PB) and BM were assessed using quantitative reverse transcriptase–polymerase chain reaction. The results of the transcript levels were depicted according to the international scale IS [22].

Response criteria were applied according to the ELN [18].

### 2.4. Statistical Analyses

Demographic characteristics are expressed as number and percentage or median for categorical and continuous variables, respectively. The differences between variables were analyzed by the ꭓ^2^ test and the Kruskal–Wallis test for categorical and continuous variables. *p* value < 5% was considered to indicate statistical significance. Overall survival (OS) and progression-free survival (PFS) were estimated according to the Kaplan–Meier method.

## 3. Results

### 3.1. Patient Characteristics

In total, 173 consecutive pediatric patients from Germany were diagnosed with CML and registered in CML-PAED-II between 1 January 2006 and 20 November 2020. Of these, 12 individuals presented with de novo BP CML during the study period. These cases were excluded from the study. The remaining 161 patients were eligible for our analyses.

The median age of patients at diagnosis was 14 years (age range, 1–17 years), and 57 patients (35%) were female and 104 patients (65%) were male. During the study period, 98 patients (61%) received imatinib treatment only, whereas 63 patients (39%) were switched from initial imatinib to second-line therapy including dasatinib or nilotinib. Patient characteristics are summarized in Table 1.

### 3.2. Incidence of Additional Non-Philadelphia Aberrations and Distribution of Cytogenetic Subgroups

An informative cytogenetic analysis from the time of diagnosis was available for 150 out of the 161 individuals (93%): 129 individuals (80%) exclusively carried the classical Philadelphia translocation t(9;22)(q34;q11) and 21 patients (13%) showed nPhAs. Of these, 12 patients (8%) carried a variant translocation, 4 patients (3%) harbored ACAs, and 5 patients (3%) had a complex karyotype (Table 1).

### 3.3. Landscape of Additional Non-Philadelphia Aberrations

In the patient group with variant translocations, we found recurrent involvement of chromosome 15 in 5 (42%) out of 12 individuals. In the remaining 7 patients, single involvement of the chromosomes 4, 5, 13, 17, 18, 19 and 22 was detected.

No recurrent abnormalities were observed in the subgroup of individuals with ACAs. An insertion ins(9;22), idic(17)(p11), trisomy 8, and del (20) (p11p13) were found in one case each. One patient with ACA also carried a variant translocation t(9;22;20).

The patients with complex karyotype exhibited various partially recurrent alterations including derivative chromosomes 1, 2, 6, 9 and 22, additional translocations t(2;9), t(2;6), t(6;22), t(2;22) and t(1;22), an additional marker chromosome, monosomy 18, trisomy 8, and trisomy 21. In addition, one individual in this cytogenetic subgroup showed an accumulation of isochromosomes i(6)(q10), i(11)(q10), i(12)(q10), i(19)(q10), and i(21)(q10).

**Figure 1:**
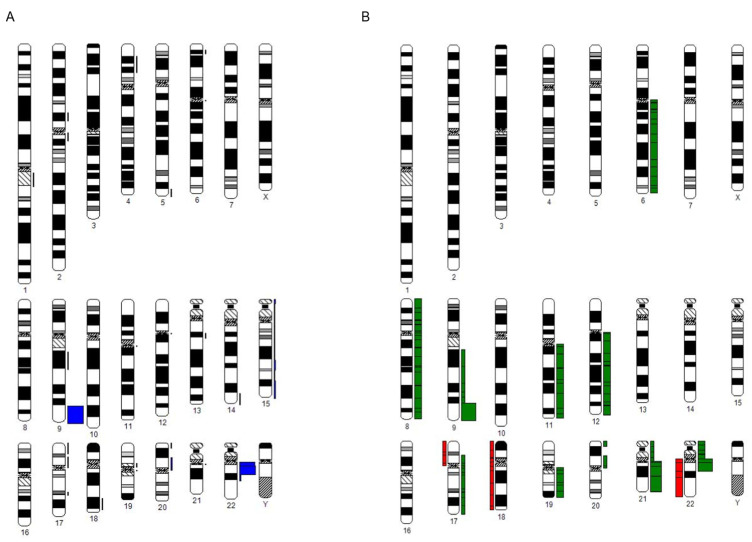
(**A**) Chromosomal distribution of cytogenetic breakpoints at diagnosis in all patients with additional non-Philadelphia aberrations (nPhAs) (cytogenetic data analysis System CyDAS). (**B**) Chromosomal distribution of gains (shown right of the chromosome in green) and losses (shown left of the chromosome in red) at diagnosis in all patients with additional non-Philadelphia aberrations (nPhAs) (cytogenetic data analysis System CyDAS).

The chromosomal breakpoints and gains and losses for all patients with additional cytogenetic abnormalities are represented in Figure 1. The karyotypes and the individual patient characteristics are listed in Table 2.

### 3.4. Clinical Presentation, Treatment, and Follow-Up

No significant differences concerning clinical or hematological parameters including the incidence of splenomegaly or levels of blood counts at diagnosis were found in patients with nPhAs compared to patients carrying only the classical Philadelphia translocation.

Overall, 8 out of 21 patients with nPhAs (38%) were switched from imatinib to dasatinib treatment. This proportion was comparable to the whole patient cohort, where 63 out of 161 patients (39%) were switched from imatinib to dasatinib (Table 1). Based on our data, the eight patients with nPhAs were switched due to suboptimal response according to the ELN criteria and intolerance had not been documented in these cases.

A total of 6 of the 12 patients with variant translocations received first-line therapy only with imatinib during the study period, 5 patients were switched to second-line therapy with dasatinib within 3–36 months after initiation of treatment and 1 patient of this subgroup underwent upfront allogeneic hematopoietic stem cell transplantation (HSCT) 3 months after diagnosis.

Of the patients carrying ACAs, three remained on imatinib throughout the study period and one was switched to dasatinib 6 months after the start of therapy.

In the group of patients with complex karyotype, one was treated with imatinib only, two were switched to dasatinib after 12 and 18 months, respectively, one individual received preemptive HSCT after 6 months and one developed secondary BP 17 months after initial diagnosis and deceased after HSCT (Table 2).

The median time of follow-up was 53 months (range 6–139) for patients with variant translocations, 68 months (range 46–77) for patients with ACAs, and 42 months (range 6–98) for patients with complex karyotype.

### 3.5. Impact of Cytogenetic Findings on Cytogenetic and Molecular Response Characteristics

**Figure 2:**
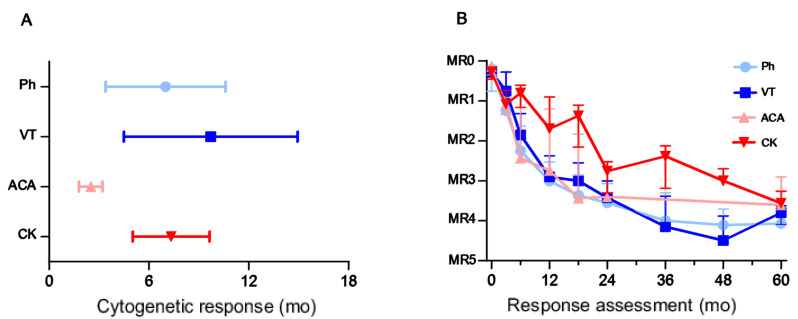
(**A**) Time point of cytogenetic response in patients carrying only the *BCR::ABL1* translocation (Ph) compared to patients with variant translocations (VT), additional chromosomal aberrations (ACA) and complex karyotype (CK). (**B**) Initial *BCR::ABL1* transcript level and treatment response over time in patients carrying only the *BCR::ABL1* translocation (Ph) compared to patients with variant translocations (VT), additional chromosomal aberrations (ACA) and complex karyotype (CK).

No differences were observed when comparing the time point of cytogenetic response in individuals exhibiting only the classical translocation t(9;22)(q34;q11) and patients harboring a variant translocation or a complex karyotype. In the group of patients with ACAs, shorter intervals between diagnosis and cytogenetic response were found (Figure 2A).

Correlating the cytogenetic subtype with *BCR::ABL1* transcript levels during therapy, no differences were seen between the group of patients with the classical translocation t(9;22)(q34;q11) only and patients with variant translocation or ACAs. The individuals with complex karyotype at diagnosis exhibited a trend to inferior treatment response (Figure 2B).

### 3.6. Impact of Cytogenetic Findings on Survival

In this cohort, seven patients progressed to secondary BP CML after a median of 8 months (range 2–17). Six of these individuals carried only the classical translocation t(9;22)(q34;q11) and one harbored a complex karyotype at diagnosis. Two deaths were reported. One death occurred in the group of patients harboring only the classical translocation t(9;22)(q34;q11) at diagnosis and one in the group of patients with complex karyotypes.

**Figure 3:**
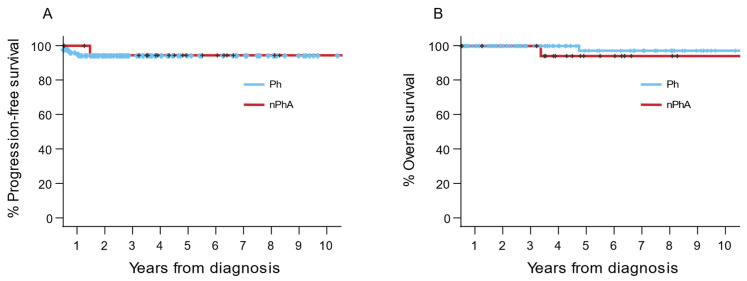
(**A**) Progression-free survival in patients carrying only the *BCR::ABL1* translocation (Ph) compared to all patients with additional non-Philadelphia aberrations (nPhAs). (**B**) Overall survival in patients carrying only the *BCR::ABL1* translocation (Ph) compared to all patients with additional non-Philadelphia aberrations (nPhAs).

The PFS in this cohort is depicted in Figure 3A. The probability of PFS at 10 years was 94.6% (95% CI, 90.5–98.4%) for the patients with the classical translocation t(9;22)(q34;q11) and 95.2% (95% CI, 86.2–95.9%) for the patients with nPhAs. The OS probability at 10 years in this cohort, shown in Figure 3B, was 99.2% (95% CI, 95.7–98.7%) for the patients with the classical translocation t(9;22)(q34;q11) and 95.2% (95% CI, 88.6–96.6%) for the patients with nPhAs. As a result, there were no differences in PFS and OS in the patients with nPhAs compared to the patients with the classical translocation t(9;22)(q34;q11).

## 4. Discussion

In adult patients with CP CML, nPhAs have been comparably well investigated and occur in 10 to 12% of cases at diagnosis [4,23]. Among these changes are variant translocations observed in 5% to 10% of newly diagnosed patients involving one or more chromosomes in addition to t(9;22) implicating another breakpoint. In adults with CML, variant translocations are not anymore considered to bear an adverse prognostic impact [24,25].

Additional chromosomal aberrations, detected in a minority of patients (5%), have been classified based on their frequency as “major” and “minor” route [2,3]. The most commonly observed “major” route abnormalities found in >10% of cases with ACAs are trisomy 8, an additional Philadelphia chromosome (Ph), i(17)(q10), and trisomy 19. Because this classification system does not consider the impact of the alterations on treatment response and prognosis, Wang et al. more recently introduced a prognostically oriented revision of the classification of ACAs. They identified two patient groups. The first carried trisomy 8, –Y, and an extra Ph with a comparably good response and better survival. The second group harbored i(17)(q10), −7/del(7q), and 3q26.2 rearrangements associated with an inferior response to treatment and survival [26].

Increasing evidence has accumulated on a different pathogenetic background of CML in children and adolescents compared to adults [7,8,9,10,11,12,13,14]. This could potentially also include a variable cytogenetic landscape of pediatric CML. However, owing to the rarity of CML in childhood and adolescence, the knowledge on nPhAs in pediatric CML is still highly limited. Millot and colleagues have published data on nPhAs in a cohort of 301 patients, who had been enrolled in the I-CML-Ped study [15]. They identified 19 individuals (6.3%) presenting with nPhAs at diagnosis: 5 children (1.7%) had a variant t(9;22) translocation, 13 children (4.3%) had ACAs, and 1 had both. In their study, Millot et al. observed no impact of variant translocations or ACAs on progression-free and overall survival and they conclude that these changes observed at diagnosis could not be considered an adverse prognostic factor in children and adolescents with CP CML.

Based on the data of the German national CML-PAED-II-study and following registry, we observed some considerable differences compared to the report from the I-CML-Ped study.

Firstly, we identified a significantly higher proportion of 21 out of 161 patients (13%) with initial nPhAs in this pediatric cohort than described in the I-CML-Ped study. Moreover, the distribution patterns of the cytogenetic subgroups were different and, in our analysis, we found variant translocations being more frequent with 12 patients (8%) than ACAs with 4 patients (3%). Moreover, we identified a small group of five patients (3%) exhibiting a complex karyotype at time of diagnosis. Thus, compared to the study by Millot et al., the incidence of nPhAs and the distribution of cytogenetic subgroups found in this cohort corresponded more closely to the data from adult patients with CML, where 5–10% of cases with variant translocation [24,27,28] and 5% of ACAs [2,3,26] have been reported.

One reason for these discrepancies between the report of the I-CML-Ped study and this analysis concerning the percentage of patients with nPhAs and the proportional distribution of the subgroups with variant translocations, ACAs and complex karyotype could be the overall still limited total number of included patients. A possible further explanation and strength of our approach is that we here assessed unique data of patients consecutively enrolled in the German national CML-PAED-II study matched with the German national childhood cancer registry, whereas the I-CML-Ped study incorporates data from varying countries worldwide. Including a nearly complete set of cytogenetic analyses performed exclusively in certified laboratories at diagnosis in 93% of cases, our data are therefore likely to be representative. Because the I-CML-Ped study comprises cytogenetic results obtained in various different laboratories, one cannot exclude that a technical heterogeneity might also have contributed to the in part discrepant cytogenetic findings

Concerning the landscape of nPhAs, we discovered recurrent involvement of chromosome 15 in variant translocations in 5 of the 12 patients (42%). Of the most common nPhAs seen in adults [26,29,30] (>10% of cases with ACAs), trisomy 8, an extra Philadelphia chromosome (Ph), i(17)(q10), and trisomy 19, only trisomy 8 was encountered in two cases and idic17(p11) in one case in this analysis. These two particular alterations did not principally differ from the same aberrations found in adult patients. However, based on our data, they appear to be less frequent in pediatric patients and instead, we observed various other partially recurrent alterations including derivative chromosomes 1, 2, 6, 9 and 22, additional translocations (t(2;9), t(2;6), t(6;22), t(2;22), t(1;22)), additional marker chromosome, monosomy 18, trisomy 21, and different isochromosomes (i(6)(q10), i(11)(q10), i(12)(q10), i(19)(q10), i(21)(q10)), which are not described to occur frequently in adult patients with CML. These findings could suggest a distinct cytogenetic landscape in this pediatric cohort and thus once more highlight differences in the biology of pediatric CML.

Corresponding to the findings of the I-CML Ped study, patients presenting with either variant translocation or ACAs at diagnosis showed no differences of molecular response to TKI treatment or survival. However, the small subgroup of patients with complex karyotype identified in our analysis was the only exhibiting a trend to inferior molecular response. This latter observation would also be in line with data from adult patients with CML where the concurrence of two or more nPhAs conferred an adverse outcome [26]. It appears conceivable, that these patients might benefit from an early switch to second-line TKI therapy.

## 5. Conclusions

This evaluation provides a detailed insight into the cytogenetic landscape of pediatric CML and reveals also differences compared to the previous report from the I-CML-Ped study and analyses in adult patients with CML. Considering the possible prognostic role of the occurrence of two or more nPhAs at diagnosis, our data underline the general significance of cytogenetic analysis in pediatric CML.

The numbers of pediatric patients with CML assessed in this and previous studies are still too low to allow general conclusions. Therefore, joint international efforts to increase the value of future studies enabling a deep and individual analysis of the potential role and relationship of nPhAs in pediatric CML are required.

## Figures and Tables

**Table 1:** Cohort characteristics of *n* = 161 pediatric patients of the CML-PAED-II-Cohort diagnosed in chronic phase CML.

**Demographic Data**	
Age, median years (range)	14 (1–17)
Sex (f/m)	57/104
**Hematology**	
Leukocytes (10E9/L), median (range)	189 (6–1038)
Platelets (10E9/L), median (range)	504 (102–2845)
**Rearrangement/Transcript**	
e13/a2 (%)	59 (37)
e14/a2 (%)	82 (51)
e13/a2 and e14/a2 (%)	14 (9)
No data (%)	6 (4)
**Cytogenetics**	
*BCR::ABL1* only (%)	129 (80)
Variant translocation (%)	12 (8)
Additional chromosomal abnormalities (%)	4 (3)
Complex karyotype (%)	5 (3)
No data (%)	11 (7)
**Treatment**	
Imatinib only (%)	98 (61)
Switch to dasatinib/nilotinib (%)	63 (39)

**Table 2:** Individual characteristics of *n* = 21 patients with variant translocations (VT), additional chromosomal aberrations (ACA) and complex karyotype (CK). A minimum of 15 metaphases were analyzed in each patient.

UPN.	Group	Age (y)	Sex	Cytogenetics at Diagnosis	Treatment/Course	Best MR	Follow-Up (mo)
1	VT	14	f	46,XX,t(9;22;18)(q34,q11;q22)/46,XX	Imatinib	MR6	98
2	VT	5	m	46,XY,t(5;9;22)(q35;q34;q11.2)	Imatinib, HSCT	MR6	14
3	VT	10	m	46,XY,t(9;22;15)(q34;q11;p13)	Imatinib	MR4	79
4	VT	17	m	46,XY,t(9;22;15)(q34;q11;?)	Imatinib→Dasatinib	MR4	65
5	VT	3	f	46,XX,t(9;22;15)(q34;q11;q22)	Imatinib	MR6	137
6	VT	9	m	46,XY,t(9;22;15)(q34;q11;q26)	Imatinib→Dasatinib	MR4	51
7	VT	11	f	46,XX,t(9;22;19)(q34;q11;p12)	Imatinib	MR4.5	41
8	VT	14	m	46,XY,der(22)t(9;22)(q34;q11)	Imatinib→Dasatinib	MR6	57
9	VT	10	f	46,XX,t(9;22;15)(q34;q11;q25)	Imatinib→Dasatinib	MR3	53
10	VT	14	m	46,XY,t(9;22;13)(q34;q11;q13)	Imatinib	MR4	46
11	VT	11	m	46,XY,t(9;22;17)(q34;q11;q22)	Imatinib→Dasatinib	MR4	38
12	VT	6	m	46,XY,t(4;9;22)(p15;q34;q11)	Imatinib	MR2	6
13	ACA	12	f	46,XX,ins(9;22)(q34;q11q12),t(14;22;17) (q32;q21;p13)/46,XX	Imatinib	MR6	58
14	ACA	5	f	46,XX,t(9;22)(q34;q11)/46,idem,idic(17)(p11)	Imatinib	MR6	96
15	ACA	13	f	46,XX,t(9;22)(q34;q11)/47,idem,+8	Imatinib	MR6	74
16	ACA	13	m	46,XY,der(9)t(9;22;20)(q34;q11;p11)del(20) (p11p13),der(22)t(9;22)(q34;q11)	Imatinib→Dasatinib	MR1	45
17	CK	13	f	46,XX,der(2)t(2;9)(p12;q21)t(2;6)(q11;p24)t(9;22) (q34;q11),der(6)t(6;22)(p24;q11)t(9;22)(q34;q11),der(9) t(2;9)(p12;q25),der(22)t(2;22)(q11;q11)	Imatinib→Dasatinib	MR6	72
18	CK	3	m	46,XY,der(1)t(1;22)(q12;q11)t(9;22)(q34;q11), der(9)t(9;22)(q34;q11),der(22)t(1;22)(q12;q11)	Imatinib→Dasatinib	MR3	96
19	CK	15	m	46,XY,t(9;22)(q34;q11/46,idem,-18,+mar[cp9]	Imatinib	MR4	42
20	CK	16	m	46~53,XY,+i(6)(q10),t(9;22)(q34;q11)x2,+i(11)(q10), +i(12)(q10),+i(19)(q10),+i(21)(q10),+der(22)t(9;22) (q34;q11) (cp8)	Imatinib→BP, death after HSCT	MR6	40
21	CK	17	m	49,XY,+8,t(9;22)(q34;q11),+21,+der(22)t(9;22) /46,XY	Imatinib, HSCT	MR0	6

## Data Availability

The data presented in this study are available only on request from the corresponding author. The data are not publicly available due to privacy and ethical restrictions.
